# Association between *SLCO1B1* genetic polymorphisms and bleeding risk in patients treated with edoxaban

**DOI:** 10.1038/s41598-023-43179-7

**Published:** 2023-09-25

**Authors:** Ji Min Han, Eun Jeong Jang, Jeong Yee, Tae-Jin Song, Dong-Hyeok Kim, Junbeom Park, Hye Sun Gwak

**Affiliations:** 1 College of Pharmacy, Chungbuk National University, Cheongju-Si, Korea; 2 College of Pharmacy and Graduate School of Pharmaceutical Sciences, Ewha Womans University, 52 Ewhayeodae-Gil, Seodaemun-Gu, Seoul, 03760 Republic of Korea; 3 Department of Neurology, Ewha Womans University Seoul Hospital, Ewha Womans University College of Medicine, Seoul, Korea; 4 Department of Cardiology, Ewha Womans University Seoul Hospital, Ewha Womans University College of Medicine, Seoul, Korea; 5 Division of Cardiology, Department of Internal Medicine, Ewha Womans University Mokdong Hospital, Ewha Womans University College of Medicine, Seoul, 07985 Korea

**Keywords:** Cardiology, Health care, Medical research, Risk factors

## Abstract

Since *SLCO1B1* encodes the uptake transporter OATP1B1, which can influence the pharmacokinetic and pharmacodynamic profiles of edoxaban, polymorphisms in *SLCO1B1* may affect the edoxaban response. This study aimed to investigate the association between *SLCO1B1* gene polymorphisms and the bleeding risk in patients receiving edoxaban. We genotyped 10 single-nucleotide polymorphisms (SNPs) from the *SLCO1B1* gene in patients receiving edoxaban. We also analyzed rs3842 of *ABCB1* as a confounder. The odds ratio (OR) and adjusted OR (AOR) were calculated from univariate and multivariable analysis, respectively. The area under the receiver operating characteristic curve (AUROC) was constructed for the discrimination of the model. A total of 159 patients receiving edoxaban were analyzed. Overdose and rs4149056 showed significant association with bleeding complications by around 11- and 5.5-fold, respectively. Additionally, patients with the rs4149057 variant allele (C) had a 3.9-fold increased bleeding risk compared with wild-type homozygote carriers (TT), whereas rs2306283 variant homozygote (GG) carriers had a 0.27-fold reduced bleeding risk compared with wild-type allele (A) carriers. Patients with the variant-type homozygote (CC) of *ABCB1* rs3842 had a higher bleeding risk than T allele carriers (AOR = 5.3 and 5.9). The final models for multivariable analyses were acceptable based on the AUROC values (> 0.70). These findings may help predict bleeding risk in patients taking edoxaban and help personalize treatment.

## Introduction

Direct oral anticoagulants (DOACs) are gradually increasing in use due to their rapid anticoagulant effect and similar efficacy to vitamin K antagonists^1^. Despite the advantage of not requiring a routine international normalized ratio test, bleeding including gastrointestinal bleeding, hemorrhage, hematochezia, hematuria, and epistaxis is a major complication of DOAC use^2^. Especially, Asian individuals with DOACs showed a higher risk of bleeding, including intracranial hemorrhage than non-Asians and may require individualized DOAC treatment^3^.

Several studies in pharmacogenomics have focused on individualizing drug treatment with DOACs, primarily investigating genes associated with drug metabolism and transport. For example, in the case of dabigatran, which is the only DOAC that is not metabolized by CYP P450, it has been reported that the *CES1* gene polymorphisms were associated with a lower dabigatran concentration due to the significant role of CES1 activity in drug metabolism^4,5^. For rivaroxaban, the impact of *ABCB1* gene polymorphism on its clinical significance remains inconclusive. While rs2032582 homozygous mutated genotype was associated with rivaroxaban-induced hemorrhage^6^, rs2032582 was not associated with rivaroxaban pharmacokinetic parameters in healthy volunteers^7^. In the case of apixaban, rs2231142 of *ABCG2* gene^8^, and rs776746 of *CYP3A5* were associated with increased plasma concentrations^9^. Conversely, there is limited research on pharmacogenomics studies focusing on edoxaban. Candidate genes involved in edoxaban metabolisms, such as *CES1, CYP3A4/5, ABCB1,* and *SLCO1B1*, have been considered, but only the relationship of rs1045642 of *ABCB1* gene and rs4149056 and rs2306283 of *SLCO1B1* gene with the pharmacokinetic parameters of edoxaban was studied^10,11^.

*SLCO1B1* encodes the uptake transporter OATP1B1, which can influence the pharmacokinetic and pharmacodynamic profiles of its substrates^12–14^. Several studies have investigated that various SNPs in *SLCO1B1* affect the pharmacokinetic profiles of medications in various diseases. Statins are among the most studied drugs for their association with *SLCO1B1* polymorphism. In a simvastatin PK study conducted on healthy volunteers, participants with the *SLCO1B1* rs4149056 CC genotype had a 221% higher AUC_0–∞_ of simvastatin acid than those with rs4149056 TT genotype^15^. In a lovastatin study, SLCO1B1*5/15 or *15/*15 genotype group showed about threefold increased AUC_0-24_ compared to the SLCO1B1*1A/*1A genotype group^16^. In addition, it was known that the *SLCO1B1* c.463CA genotype was associated with about 40% lower rifampin concentration in Tuberculosis patients^17^. The relationship between the increased AUC of lopinavir and rs4149056 in HIV-infected children was also reported^18^.

Currently, the impact of *SLCO1B1* polymorphism on OATP1B1 substrates is mainly studied in relation to edoxaban, specifically focused solely on the variants rs4149056 and rs2306283^10,11^. Moreover, these studies have primarily involved pharmacokinetic analyses, with no exploration of the pharmacodynamic effects of *SLCO1B1* polymorphism on edoxaban.

There are many ways to select SNPs in genetic studies. Linkage disequilibrium (LD) plays a critical role in genome-wide association studies for identifying genetic variation^19^. Genotyping of tag SNPs representing LD blocks is known to be sufficient to capture most haplotype structures of the human genome^20,21^. Therefore, the SNP selection process using tag SNPs can contribute to finding important SNPs more accurately. Meanwhile, *ABCB1* rs3842, a 3’-UTR SNP, can affect protein elimination by destroying or creating miRNA binding sites^22^. In addition, *ABCB1* rs3842 was found to affect the incidence of bleeding events in patients with DOACs in our previous study^23^. Adjusting it as a confounder is necessary to reveal more accurate effects of *SL*C*O1B1* SNPs.

Therefore, the objective of this study was to investigate the association between various *SLCO1B1* gene polymorphisms and the risk of bleeding complications in patients on edoxaban.

## Materials and methods

### Study patients and data collection

This study was a retrospective analysis of prospectively collected samples from June 2018 to December 2021. We recruited patients who had been using edoxaban and retrospectively collected the patients’ previous records. We collected samples with patient consent on the day of the patient’s first outpatient visit after the start of the study. It was conducted at Ewha Womans University Mokdong Hospital and Ewha Womans University Seoul Hospital.

The study subjects were individuals aged ≥ 20 years old who received edoxaban. Patients were excluded if they met the following criteria: (1) had thromboembolic or infarction-related events during the follow-up period, (2) experienced bleeding that was minor or unverified by health professionals while on treatment, (3) experienced bleeding after one year of edoxaban therapy, or (4) treated with edoxaban for less than three months (in the control group). We followed up for bleeding for one year after edoxaban initiation. The primary endpoint was any one-year major bleeding event and clinically relevant non-major bleeding (CRNMB) according to the International Society on Thrombosis and Haemostasis (ISTH) criteria^24,25^.

Among the study population, patients who experienced any one-year major bleeding event or CRNMB were classified as a case group and other patients were classified as a control group.

We obtained the data from electronic medical records. We collected patient demographic data, including sex, age, body mass index, creatinine clearance, prescription dose, concurrent medication, any history of myocardial infarction, stroke, transient ischemic attack, thromboembolism, or bleeding, comorbidities, smoking status, and alcohol status. The CHA_2_DS_2_-VASc stroke assessment score (congestive heart failure, hypertension, age ≥ 75 years, diabetes mellitus, stroke, vascular disease, age 65–74 years, and sex category; range 0–9) was calculated from its component variables^26^. The modified HAS-BLED (hypertension, abnormal renal or liver function, stroke, bleeding history or predisposition, elderly (age ≥ 65 years), concomitant drug and alcohol use; range 0–8) score, which is a specific risk score designed for bleeding risk assessment, were calculated from its component variables^27^.

The studies involving human participants were reviewed and approved by the Institutional Review Board of Ewha Womans University Mokdong Hospital and Ewha Womans University Seoul Hospital in accordance with the 1975 Helsinki Declaration and its later amendments (IRB numbers 2018-04-006 and 2019-05-038, respectively). Written informed consents from all participants were obtained before enrollment.

### Selection of SNPs and genotyping

We selected SNPs through the following process. First, the Haploreg program was used to identify minor allele frequencies and linkage disequilibrium (LD) data of each SNP in the Asian population^28^. The tagger function within the Haploview of v4.2 was used to assign tag SNPs. Tag SNPs of the *SLCO1B1* gene were assigned with a condition MAF ≥ 20% and an r^2^ threshold of 0.8 in Asian populations. Second, among them, nine SNPs, including one synonymous SNP (rs4149057^29,30^) and eight intronic SNPs (rs11045879^31,32^, rs12317268^33,34^, rs4149081^31,35^, rs999278^36^, rs2306283^13,37^, rs10841753^14,38^, rs2417957^39,40^, and rs4149042^41^) of *SLCO1B1* were selected based on previous studies and PharmGKB^42^, which is a pharmacogenomics Knowledge Base that offers information about how human genetic variation impacts drug response. Although rs4149056 (missense SNP) had a MAF of 0.13, it was included because it has been investigated for various drug-induced toxicity and studied with edoxaban^10,11,13,14,32,38^. Finally, a total of 10 SNPs were included in this study. We included *ABCB1* rs3842, a 3’-untranslated region (UTR) SNP, as a known confounder based on previous studies^23,43,44^.

The genomic deoxyribonucleic acid (DNA) of the patients was extracted from blood or saliva. Genomic DNA was extracted from EDTA blood samples using the QIAamp DNA Blood Mini Kit (QIAGEN GmbH, Hilden, Germany). Using OraGene-600 kits (DNA Genotek, Ottawa, Canada), saliva samples were collected and subjected to genomic DNA extraction with PrepIT reagents (DNA Genotek, Ottawa, Canada). The genotypes of 10 SNPs in *SLCO1B1* and *ABCB1* rs3842 were analyzed by TaqMan assay.

The TaqMan^®^ allele discrimination technique was used to perform RT-PCR on ABI 7300 instrument (Applied Biosystems, Carlsbad, CA, USA). The PCR was performed in a 25 μL optical 8-cap strip containing 11.25 μL of DNA samples and 13.75 μL of PCR mix. The PCR reagent mixture included 12.5 μL of the TaqMan Genotyping Master Mix and 1.25 μL of the 20X TaqMan SNP Genotyping Assay Mix (Applied Biosystems in Foster City, California, USA). The catalog numbers of used assays were 4,351,379 and 4,362,691. Ten minutes after denaturing at 95 °C, the PCR was run for 15 s at 92 °C for 40 cycles and 60 s at 60 °C for 40 cycles.

### Sample size

According to the RE-LY study^45^, the ROCKET-AF study^46^, and the ARISTOLE study^47^, the incidence of bleeding complications in patients taking DOAC was 14.6–18.1 per 100 person-years. Therefore, the bleeding incidence was assumed to be approximately 15.0%. It was assumed that the case group: control group = 1:5.7, a significant odds ratio of 3.5, a minor allele frequency of 0.2, and a dominant genetic model. When estimated using Quanto v.2.4 (sample size calculating program for genotyping studies) with a significance level of 5% and a power of 80%, the number of case groups required was 24 patients (number of control groups: 24 × 5.7 = 137), approximately 160 patients required. Therefore, the final target number was set at about 160 patients.

### Statistical analysis

We compared continuous variables between patients who experienced bleeding and those who did not, using unpaired t-tests. The Kolmogorov–Smirnov method was used to test for the normality of the continuous variables. We analyzed categorical variables with the chi-squared test and Fisher’s exact test. For genetic association analysis, we included both dominant and recessive models, and we selected the most appropriate model based on effect size and statistical significance. We identified independent risk factors for bleeding after adjusting for variables with P < 0.1 in the univariate analysis in addition to age, sex, and *ABCB1* rs3842 via a multivariable logistic regression model. The unadjusted odds ratio (OR) and adjusted OR (AOR) with the 95% confidence interval (CI) were calculated from univariate and multivariable analyses, respectively. For the selection of the best model, the method of backward hierarchical elimination was used. To test the fit of the prediction model, we performed the Hosmer–Lemeshow goodness-of-fit test. We further evaluated the model discrimination by calculating the area under the receiver operating characteristic curve (AUROC). The predictive power of the logistic regression model was calculated.

Time to a bleeding event was analyzed using the Kaplan–Meier survival curves and the log-rank test. The Cox proportional-hazards model was sued for the multivariable analysis. Factors having P < 0.1 from the univariate analysis along with strong confounders of sex and age were included in the multivariable analysis. Hazard ratio (HR) and adjusted HR were calculated from the univariate and multivariable analyses, respectively.

All analyses were based on two-tail statistics and were performed using the Statistical Package for Social Sciences version 20.0 (IBM Corp., Armonk, NY, USA). P < 0.05 was considered statistically significant.

## Results

**Figure 1:**
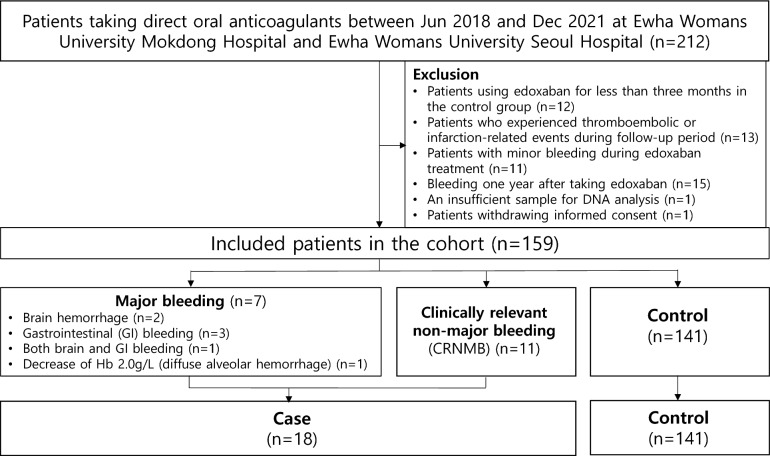
Patient flowchart.

We selected a total of 212 patients for the study, excluding 12 patients treated with edoxaban for < three months in the control group, 13 patients who experienced thromboembolic or infarction-related events during the follow-up period, 11 patients who reported minor bleeding during edoxaban treatment, 15 patients who had any bleeding at least one year after edoxaban therapy, one patient with a sample insufficient for DNA analysis, and one patient who withdrew informed consent (Fig. 1). Finally, 159 patients were included in the analysis. A total of 18 patients (11.3%) experienced bleeding complications, 7 of which were major and 11 of which were CRNMB events. The time (mean ± standard deviation) to a bleeding event was 111.72 ± 118.44 days. The indication for edoxaban treatment in the study patients was atrial fibrillation or secondary stroke prevention. Of the 18 patients, half the patients visited the emergency room or were hospitalized for bleeding. Two of them received red blood cell transfusions. All hospitalized patients recovered and were discharged.

Table 1 shows the demographic and clinical characteristics of the study population taking edoxaban. The mean age of the included patients was 71 years, and 92 patients (57.9%) were male. Approximately one-third of the patients received an underdose of edoxaban. The most common co-medications were statins, followed by beta-blockers. Approximately 97% of the patients had atrial fibrillation, and approximately 64% had hypertension. There was no significant factor for the incidence of bleeding complications.

**Table 1:** Baseline characteristics of patients who administered edoxaban.

Characteristic	No. (%) (n = 159)	Bleeding complication, No. (%) or mean ± SD		
		Bleeding	No bleeding	P
		(n = 18)	(n = 141)	
Sex				
Female	67 (42.1)	5 (27.8)	62 (44.0)	0.190
Male	92 (57.9)	13 (72.2)	79 (56.0)	
Age (years)		71.00 ± 9.89	71.05 ± 10.55	0.990
< 65	39 (24.5)	3 (16.7)	36 (25.5)	0.570
≥ 65	120 (75.5)	15 (83.3)	105 (74.5)	
BMI (kg/m^2^)		25.13 ± 3.42	24.72 ± 3.56	0.670
< 25	82 (56.6)	10 (62.5)	72 (55.8)	0.610
≥ 25	63 (43.4)	6 (27.5)	57 (44.2)	
Creatinine clearance (mL/min)		65.19 ± 16.53	70.21 ± 25.83	0.450
< 30	5 (3.4)	0 (0.0)	5 (3.8)	1.000
≥ 30	143 (96.6)	16 (100.0)	127 (96.2)	
Prescription dose^a^				
Underdose	49 (30.8)	6 (33.3)	43 (30.5)	0.080
Standard dose	106 (66.7)	10 (55.6)	96 (68.1)	
Overdose	4 (2.5)	2 (11.1)	2 (1.4)	
Co-medications				
Antiplatelets	22 (13.8)	1 (5.6)	21 (14.9)	0.470
ACEI or ARBs	80 (50.3)	6 (22.2)	74 (52.5)	0.130
Beta-blockers	97 (61.0)	13 (72.2)	84 (59.6)	0.300
Calcium channel blockers	49 (30.8)	3 (16.7)	46 (32.6)	0.170
Diuretics	36 (22.6)	4 (22.2)	32 (22.7)	1.000
Statins	100 (62.9)	9 (50.0)	91 (64.5)	0.230
CYP inducers	0 (0)	0 (0.0)	0 (0.0)	NA
CYP inhibitors	14 (8.9)	2 (11.1)	12 (8.6)	0.660
Previous myocardial infarction	14 (8.8)	1 (5.6)	13 (9.2)	0.605
Previous stroke/TIA/thromboembolism	83 (52.2)	10 (55.6)	76 (51.8)	0.762
Previous bleeding events	7 (4.4)	0 (0)	7 (5.0)	0.334
Comorbidities				
Atrial fibrillation	147 (97.4)	17 (94.4)	130 (97.7)	0.400
Hypertension	102 (64.2)	9 (50.0)	93 (66.0)	0.180
Diabetes mellitus	48 (30.2)	3 (16.7)	45 (31.9)	0.180
Heart failure	20 (12.6)	2 (11.1)	18 (12.8)	1.000
Anemia	49 (30.8)	8 (44.4)	41 (29.1)	0.180
Smoking	20 (12.6)	4 (22.2)	16 (11.3)	0.250
Alcohol	52 (37.4)	7 (43.8)	45 (36.6)	0.580
CHA_2_DS_2_-VASc risk of stroke		3.44 ± 1.72	3.81 ± 1.54	0.351
Modified HAS-BLED		2.00 ± 0.84	2.06 ± 1.00	0.796

The CHA_2_DS_2_-VAS_C_ score is a point-based system used to stratify the risk of stroke in atrial fibrillation patients. It stands for congestive heart failure, hypertension, age, diabetes mellitus, stroke, vascular disease, and sex category.

*ACEIs* angiotensin converting enzyme inhibitors, *ARBs* angiotensin II receptor blockers, *BMI* body mass index, *CYP* cytochrome P450 family, *DOACs* direct oral anticoagulants, *NA* not available, *TIA* transient ischemic attack.

^a^Standard dose was defined according to the FDA-approved labeling.

In the genotype analysis, rs4149057, rs999278, rs2306283, and rs4149056 of *SLCO1B1* and rs3842 of *ABCB1* were significantly associated with bleeding risk (Table 2).

**Table 2:** Effects of gene polymorphisms on bleeding complications in patients who administered edoxaban.

dbSNP rsID	Grouped genotype	Minor allele frequency	Amino acid change	Edoxaban		
				Bleeding	No bleeding	P
				(n = 18)	(n = 141)	
rs11045879 (T > C)	TT, CT	0.4	–	17 (94.4)	115 (81.6)	0.170
	CC			1 (5.6)	26 (18.4)	
rs12317268 (A > G)	AA, AG	0.38	–	17 (94.4)	116 (82.3)	0.188
	GG			1 (5.6)	25 (17.7)	
rs4149057 (T > C)	TT	0.27	Leu191 =	5 (27.8)	77 (54.6)	0.032
	CT, CC			13 (72.2)	64 (45.4)	
rs4149081 (G > A)	GG, AG	0.39	–	16 (94.1)	115 (81.6)	0.194
	AA			1 (5.9)	26 (18.4)	
rs999278 (C > A)	CC	0.27	–	5 (27.8)	76 (53.9)	0.037
	AC, AA			13 (72.2)	65 (46.1)	
rs2306283 (A > G)	AA, AG	0.28	Asn130Asp	13 (72.2)	67 (47.5)	0.048
	GG			5 (27.8)	74 (52.5)	
rs10841753 (T > C)	TT	0.31	–	12 (66.7)	68 (48.2)	0.141
	CT, CC			6 (33.3)	73 (51.8)	
rs2417957 (C > T)	CC	0.31	–	13 (72.2)	68 (48.6)	0.059
	CT, TT			5 (27.8)	72 (51.4)	
rs4149042 (T > C)	TT, CT	0.42	–	17 (94.4)	110 (78.0)	0.102
	CC			1 (5.6)	31 (22.0)	
rs4149056 (T > C)	TT	0.14	Val174Ala	8 (44.4)	109 (77.3)	0.003
	CT, CC			10 (55.6)	32 (22.7)	
SLCO1B1*1A carrier	Yes			13 (72.2)	64 (46.4)	0.039
	No			5 (27.8)	74 (53.6)	
SLCO1B1*1B carrier	Yes			16 (88.9)	128 (92.8)	0.563
	No			2 (11.1)	10 (7.2)	
SLCO1B1*15 carrier	Yes			10 (55.6)	31 (22.5)	0.003
	No			8 (44.4)	107 (77.5)	
*ABCB1* rs3842 (T > C)	TT, TC	0.32	–	12 (66.7)	127 (90.7)	0.003
	CC			6 (33.3)	13 (9.3)	

*ABCB1* ATP binding cassette subfamily B member 1.

We performed a multivariable logistic regression analysis using variables with P < 0.1, age, and sex. We constructed two models for the multivariable analyses of the edoxaban subgroup, as one pair of SNPs (rs4149057 and rs999278) was in LD (r^2^ = 0.97) in study population (Table 3). Model I included age, sex, overdose, rs999278, rs2306283, rs4149056, rs2417957, and rs3842. Model II included rs4149057 instead of rs999278 in Model I. Patients overdosed and variant-type allele (C) carriers of rs4149056 had an 11.0–11.4 and 5.5–5.7 fold increased bleeding risk, respectively, after adjusting for confounders. Additionally, patients with rs4149057 variant allele (C) had a 3.9-fold increased bleeding risk compared with wild-type homozygote (TT) carriers, whereas rs2306283 variant homozygote (GG) carriers had a 0.27-fold reduced bleeding risk compared with wild-type allele (A) carriers. *ABCB1* rs3842 variant homozygote carriers (CC) also showed a 5.3–5.9 fold increased bleeding risk.

**Table 3:** Univariate and multivariable regression analyses to identify predictors for bleeding complications in edoxaban users.

Predictors		Unadjusted OR	Model I	Model II	Model III	Model IV	Model V & VI
		(95% CIs)	Adjusted OR	Adjusted OR	Adjusted OR	Adjusted OR	Adjusted OR
			(95% CI)	(95% CI)	(95% CI)	(95% CI)	(95% CI)
Age	≥ 65 years	1.71 (0.47–6.27)					
Female		0.49 (0.17–1.45)					
Prescription dose	Overdose	8.69 (1.14–65.96)*	11.36 (1.23–114.36)*	11.01 (1.08–111.97)*	10.86 (1.07–110.35)*	10.82 (1.07–109.91)*	13.60 (1.59–116.64)*
rs4149057 (T > C)	CT, CC	3.13(1.06–9.24)*		3.92 (1.16–13.29)*		3.82 (1.13–12.94)*	
rs999278 (C > A)	AC, AA	3.04 (1.03–8.98)*			3.78 (1.11–12.80)*		
rs2306283 (A > G)	GG	0.35 (0.12–1.03)	0.27 (0.08–0.90)*				
rs4149056 (T > C)	TC, CC	4.26 (1.55–11.69)**	5.48 (1.75–17.23)**	5.71(1.80–18.13)**			
rs2417957 (C > T)	CT, TT	0.36 (0.12–1.07)					
SLCO1B1*15 carrier	Yes	4.32 (1.57–11.87)**			5.78 (1.82–18.36)**	5.75 (1.81–18.24)**	
SLCO1B1*1A carrier	Yes	3.006 (1.017–8.891)*					3.00 (0.96–9.40)
*ABCB1* rs3842* (T* > *C)*	CC	4.89 (1.57–15.18)**	5.89 (1.61–21.56)**	5.33 (1.45–19.55)*	5.32(1.45–19.50)*	5.28 (1.44–19.35)*	5.44 (1.66–17.86)**

Model I and III included variables of sex, age, prescription dose, *ABCB1* rs3842, *SLCO1B1* rs999278, rs2306283, rs4149056, and rs2417957. Model II included variables of sex, age, prescription dose, *ABCB1* rs3842, *SLCO1B1* rs4149057, rs2306283, rs4149056, and rs2417957. Model III included variables of sex, age, prescription dose, *ABCB1* rs3842, *SLCO1B1* rs999278, rs2417957, and SLCO1B1*15. Model IV included variables of sex, age, prescription dose, *ABCB1* rs3842, *SLCO1B1* rs4149057, rs2417957, and SLCO1B1*15. Model V included variables of sex, age, prescription dose, *ABCB1* rs3842, *SLCO1B1* rs999278, rs2417957, and SLCO1B1*1A. Model VI included variables of sex, age, prescription dose, *ABCB1* rs3842, *SLCO1B1* rs4149057, rs2417957, and SLCO1B1*1A.

*CI* confidence interval, *OR* odds ratio.

*P < 0.05, **P < 0.01.

Haplotype analyses including SLCO1B1*1A, SLCO1B1*1B, and SLCO1B1*15) were also conducted (Table 3). Among them, SLCO1B1*1A and SLCO1B1*15 were significant for bleeding events. In the multivariable analysis, patients with SLCO1B1*15 showed an increased risk of bleeding events by 5.7–5.8 folds, whereas SLCO1B1*1A failed to demonstrate statistical significance. RS999278 was a new SNP that increased the risk of bleeding complications by 3.7 times.

**Figure 2:**
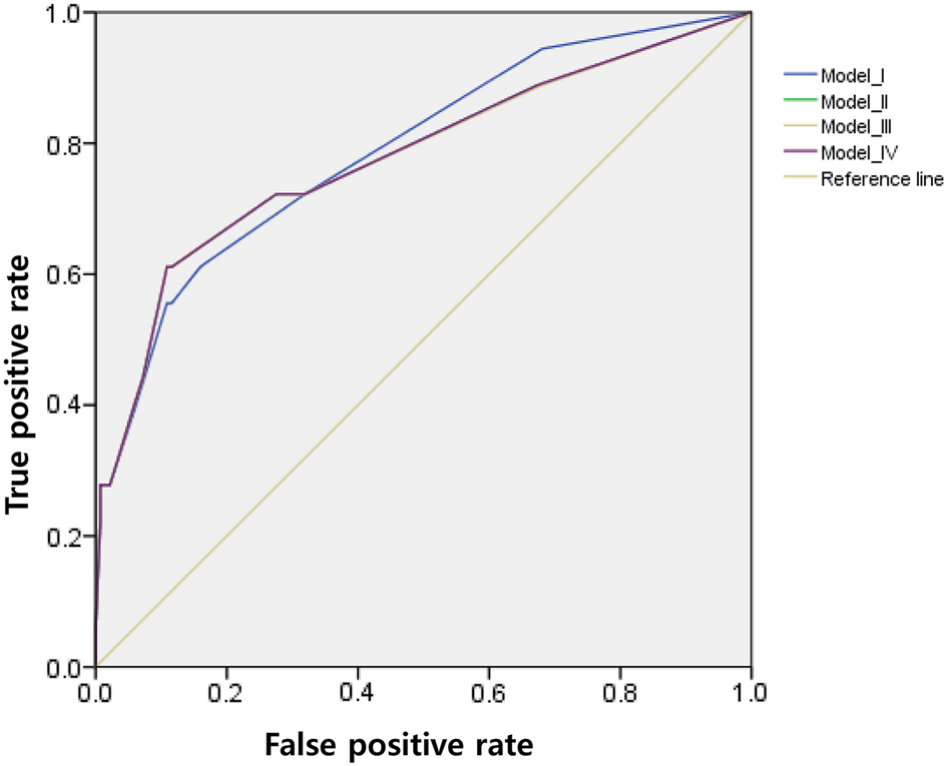
The area under the receiver operating characteristics (AUROC) curves for bleeding complications for edoxaban users.

The Hosmer–Lemeshow test for bleeding revealed a good fit for the final model (Model I: χ^2^ = 0.11, P = 0.991, Model II: χ^2^ = 2.34, P = 0.674, Model III: χ^2^ = 2.43, P = 0.657, Model IV: χ^2^ = 2.32, P = 0.677). The AUROC value (Model I: 0.79, 95% CI 0.67–0.91, Model II: 0.78, 95% CI 0.65–0.91, Model III: 0.78, 95% CI 0.64–0.91, Model IV: 0.78, 95% CI 0.65–0.91) from multivariable logistic regression indicated the acceptable performance of all models (Fig. 2). The predictive power of the bleeding prediction models was 91.1% in model I and II, 91.0% in model III and IV.

After Bonferroni correction of multiple comparisons for ten SNPs analyses, *SLCO1B1* rs4149056 and *ABCB1* rs3842 were the sole SNPs that reached statistical significance in the Table 2. When the simplest genetic model was constructed using these two SNPs, *SLCO1B1* rs4149056 and *ABCB1* rs3842 were associated with 4.7- and 5.7-fold increased bleeding risk, respectively. This genetic model still held a high predictive value (89.2%), similar to the predictive value (91.1%) of the prediction model shown initially in our study.

Since overdose can drive associations, further analyses were performed excluding overdose cases. Regardless of the model, two *SLCO1B1* SNPs (rs2306283, rs4149056) and one *ABCB1* SNP (rs3842) still showed statistical significance. Patients with the rs4149056 variant allele (C) had a 4.5-fold increased bleeding risk compared with wild-type homozygote carriers (TT), whereas rs2306283 variant homozygote (GG) carriers had a 0.25-fold reduced bleeding risk compared with wild-type allele (A) carriers. Patients with the variant-type homozygote (CC) of ABCB1 rs3842 had a higher bleeding risk than T allele carriers (AOR = 5.7).

Time to a bleeding event was analyzed. Among the clinical characteristics, there were no factors affecting the time to bleeding events (Supplementary Table 1). For genetic factors, *SLCO1B1* rs4149056, rs4149057, rs999278 and *ABCB1* rs3842 were significantly related with time to bleeding events in both univariate and multivariable analyses (Supplementary Tables 2 and 3).

Two models were constructed for the multivariable analyses, as one pair of SNPs (rs4149057 and rs999278) was in LD (r^2^ = 0.97) in the study population (Supplementary Table 3). Rs4149056 and rs4149057 were associated with an increased hazard of time to bleeding by around 3.9 times and 3.0 times, respectively. Patients with the rs999278 variant allele (A) had a 2.9-fold increased hazard of time to bleeding compared with wild-type homozygote carriers (CC). Patients with the variant-type homozygote (CC) of *ABCB1* rs3842 had a 3.7–3.8- times the hazard of time to bleeding compared to T allele carriers.

## Discussion

This study revealed the clinical and genetic risk factors associated with bleeding complications during edoxaban therapy. Edoxaban overdose, *SLCO1B1* rs4149057, rs2306283, and rs4149056, and *ABCB1* rs3842 were significant factors for bleeding risk.

OATP1B1, a drug transporter expressed in the liver, plays an important role in transporting drugs and endogenous substrates from the blood into the hepatocytes^48^. The *SLCO1B1* gene polymorphism, which encodes OATP1B1, may affect transporter activity. Previous studies on the association between *SLCO1B1* and DOACs have been mainly limited to edoxaban^10,11^.

Among the *SLCO1B1* SNP rs4149057, rs2306283, and rs4149056 that were significant in our edoxaban study, rs4149056 is the only SNP whose association with edoxaban has been studied. In the case of rs4149056, the relationship with edoxaban pharmacokinetics was investigated, but the relationship with edoxaban pharmacodynamics including bleeding has not yet been studied.

Rs4149056 (SLCO1B1*5) is a functional polymorphism in exon 5 and a well-studied SNP associated with drug toxicities such as stain-induced myopathy and methotrexate toxicity^13,14^. Rs4149056 was typically associated with reduced transporter activity, resulting in increased systemic drug exposure and increased toxicity risk^13,38^. In our study, rs4149056 significantly increased the bleeding risk. A pharmacogenomic analysis that combined 14 phase 1 studies investigated the relationship between *SLCO1B1* gene polymorphism (rs4149056) and edoxaban pharmacokinetics^10^. Although the effect of rs4149056 on overall edoxaban exposure was insignificant, carriers with variant allele (C) showed an increased exposure to M4, the most-abundant metabolite of edoxaban. M4 was considered an insignificant metabolite for the overall anticoagulant effect because it accounted for less than 10% of the total anticoagulant exposure^49^. In another edoxaban study on SLCO1B1*15 haplotype, the correlation between prothrombin time and M4 concentration was significant in non-valvular atrial fibrillation patients with repeated administration, and the clinical contribution of M4 was confirmed^11^.

Unlike rs4149056, studies on effects of rs4149057 and rs2306283 in patients taking edoxaban are limited. In the case of rs2306283, the relationship between rs2306283 and statin-induced myopathy has been primarily investigated. However, the role of rs2306283 polymorphism in transport remains controversial. The variant allele (G) of rs2306283 was not associated with statin-induced myopathy^50^, but in another study, SLCO1B1*15 carriers (rs4149056 allele C and rs2306283 allele G) showed higher rosuvastatin plasma concentration than *SLCO1B1* haplotype (rs4149056 allele T and rs2306283 allele A), which may increase the risk of statin-induced myopathy^51^. Although rs4149057 is a synonymous SNP located in exon 5 of *SLCO1B1*, it is associated with the clearance change of mitotane and irinotecan, substrates of OATP1B1^29,30^. This study suggests that they play a critical role in edoxaban therapy. Future studies should elucidate the mechanisms of these SNPs.

The *ABCB1* gene, which encodes the P-gp efflux pump, has been one of the frequently studied genes^52^. In our previous pharmacogenomics study, we found *ABCB1* rs3842 was a significant factor associated with increased bleeding risk in patients with DOACs^23^. *ABCB1* rs3842 is a 3’-UTP SNP that might influence protein expression by disrupting or creating microRNA binding sites^22^. Previous research also have shown that carriers of the *ABCB1* rs3842 variant allele (C) had high bioavailability or lower disease activity scores^43,44^. Accordingly, we analyzed including *ABCB1* rs3842 as a confounder, and rs3842 was also found to be a factor affecting bleeding complications in this study.

In this study, we observed an elevated bleeding risk among patients who experienced an overdose of edoxaban. To determine edoxaban overdose, we considered dose adjustment criteria based on the patient's renal function. DOAC overdose increases the risk of bleeding events^53^. In a systematic review regarding off-label dosages of DOACs, the incidence of adverse reactions was higher in patients administered an overdose than in those administered the recommended dose^54^. DOAC overdosing including edoxaban was associated with not only bleeding events but also stroke/systematic embolism, all-hospitalization, and all-cause mortality.

Several limitations were observed in this study, including relatively small sample size and exclusive focus on Asian individuals residing in Korea, which could potentially restrict the generalizability of the findings. Although we collected samples prospectively, there is a risk of bias associated with a retrospective design because patient data were collected retrospectively. Furthermore, the follow-up period was limited to one year to eliminate the likelihood of non-drug-related bleeding. Since this study was conducted on edoxaban, a substrate of *SLCO1B1*, the results of this study cannot be applied to other drugs or healthy populations. However, to our knowledge, this is the first study to investigate the association between *SLCO1B1* polymorphisms and edoxaban-related bleeding complications. The results must be validated in different populations to generalize and apply them in clinical practice.

### Supplementary Information

Supplementary Tables.

## Data Availability

The datasets used and/or analysed during the current study available from the corresponding author on reasonable request.
