# Supplementary material for: Association between SLCO1B1 genetic polymorphisms and bleeding risk in patients treated with edoxaban
Source: Sci Rep. 2023 Sep 25;13:15967. doi: 10.1038/s41598-023-43179-7 (PMC10520058; doi:10.1038/s41598-023-43179-7)
Supplement: Supplementary file 1 — Supplementary Tables. [file 41598_2023_43179_MOESM1_ESM.docx]

Supplementary Table 1. Time to bleeding complications related to edoxaban administration in baseline characteristics

| Characteristic | No. (%) | Time to bleeding complications  mean (95% CI) | P |
| --- | --- | --- | --- |
|  | (n= 159) |  |  |
|  |  |  |  |
| **Sex** |  |  | 0.192 |
| Female | 67 (42.1) | 346.0 (329.0-363.1) |  |
| Male | 92 (57.9) | 329.3(309.0-350.0) |  |
| **Age (years)** |  |  | 0.433 |
| < 65 | 39 (24.5) | 339.9(312.6-367.3) |  |
| ≥ 65 | 120 (75.5) | 335.2(319.1-351.2) |  |
| **BMI (kg/m^2^)** |  |  | 0.634 |
| < 25 | 82 (56.6) | 338.9(321.0-356.7) |  |
| ≥ 25 | 63 (43.4) | 337.7(316.1-359.4) |  |
| **Creatinine clearance** (mL/min) |  |  | 0.442 |
| < 30 | 5 (3.4) | - |  |
| ≥ 30 | 143 (96.6) | - |  |
| **Prescription dose^a^** |  |  | 0.050 |
| Underdose | 49 (30.8) | 339.6(326.3-352.8) |  |
| Standard dose | 106 (66.7) |  |  |
| Overdose | 4 (2.5) | 211.0 (60.0-362.0) |  |
| **Co-medications** |  |  |  |
| Antiplatelets | 22 (13.8) | 349.1(318.6-379.6) | 0.299 |
| ACEI or ARBs | 80 (50.3) | 347.0(331.7-362.2) | 0.127 |
| Beta-blockers | 97 (61.0) | 332.8(314.3-351.4) | 0.318 |
| Calcium channel blockers | 49 (30.8) | 345.5(324.1-367.0) | 0.178 |
| Diuretics | 36 (22.6) | 337.0(308.6-365.3) | 0.976 |
| Statins | 100 (62.9) | 346.3(332.2-360.4) | 0.214 |
| CYP inducers | 0 (0) | - |  |
| CYP inhibitors | 14 (8.9) | 322.9(268.9-376.9) | 0.733 |
| **Previous myocardial infarction** | 14 (8.8) | 364.6(363.8-365.4) | 0.583 |
| **Previous stroke/TIA/thromboembolism** | 83 (52.2) | 338.8(320.8-356.9) | 0.794 |
| **Previous bleeding events** | 7 (4.4) | - | 0.348 |
| **Comorbidities** |  |  |  |
| Atrial fibrillation | 147 (97.4) | 336.1(321.7-350.5) | 0.384 |
| Hypertension | 102 (64.2) | 342.0(326.6-357.5) | 0.194 |
| Diabetes mellitus | 48 (30.2) | 351.6(335.3-367.8) | 0.183 |
| Heart failure | 20 (12.6) | 338.0(300.8-375.2) | 0.850 |
| **Anemia** | 49 (30.8) | 323.7(294.6-352.7) | 0.184 |
| **Smoking** | 20 (12.6) | 311.5(255.9-367.0) | 0.158 |
| **Alcohol** | 52 (37.4) | 333.4(308.4-358.4) | 0.579 |
| ACEIs, angiotensin converting enzyme inhibitors; ARBs, angiotensin II receptor blockers; BMI, body mass index; CYP, cytochrome P450 family; DOACs, direct oral anticoagulants; NA, not available; TIA, transient ischemic attack The CHA2DS2-VASC score is a point-based system used to stratify the risk of stroke in atrial fibrillation patients. It stands for congestive heart failure, hypertension, age, diabetes mellitus, stroke, vascular disease, and sex category. ^a^Standard dose was defined according to the FDA-approved labeling. | | | |

Supplementary Table 2. Time to bleeding complications related to edoxaban administration in *SLCO1B1* grouped genotypes

| dbSNP rsID | Grouped genotype | No. (%) | Time to bleeding  mean (95% CI) | P |
| --- | --- | --- | --- | --- |
|  |  | (n= 159) |  |  |
|  |  |  |  |  |
| rs11045879 (T>C) | TT, CT | 132(83.0) | 332.8(316.7-348.8) | 0.178 |
|  | CC | 27(17.0) | 353.7(332.1-350.2) |  |
| rs12317268 (A>G) | AA, AG | 133(83.6) | 333.0(317.1-348.9) | 0.196 |
|  | GG | 26(16.4) | 353.3(330.8-375.8) |  |
| rs4149057 (T>C) | TT | 82(51.6) | 351.6(338.1-365.2) | 0.031 |
|  | CT, CC | 77(48.4) | 320.1(295.9-344.2) |  |
| rs4149081 (G>A) | GG, AG | 131(82.9) | 332.6(316.4-348.7) | 0.201 |
|  | AA | 27(17.1) | 353.7(332.1-375.4) |  |
| rs999278 (C>A) | CC | 81(50.9) | 351.4(337.7-365.2) | 0.036 |
|  | AC, AA | 78(49.1) | 320.6(296.7-344.5) |  |
| rs2306283 (A>G) | AA, AG | 80(50.3) | 324.7(302.4-346.9) | 0.051 |
|  | GG | 79(49.7) | 348.2(332.1-364.2) |  |
| rs10841753 (T>C) | TT | 80(50.3) | 324.4(301.5-347.3) | 0.136 |
|  | CT, CC | 79(49.7) | 348.4(333.4-363.4) |  |
| rs2417957 (C>T) | CC | 81(51.3) | 320.7(296.9-344.5) | 0.055 |
|  | CT, TT | 77(48.7) | 352.4(339.5-365.3) |  |
| rs4149042 (T>C) | TT, CT | 127(79.9) | 331.5(314.9-348.1) | 0.108 |
|  | CC | 32(20.1) | 355.5(337.2-373.8) |  |
| rs4149056 (T>C) | TT | 117(73.6) | 345.0(331.0-359.0) | 0.003 |
|  | CT, CC | 42(26.4) | 312.2(278.2-346.1) |  |
| *ABCB1* rs3842 (T>C) | TT, TC | 139(88.0) | 343.0(329.8-356.1) | 0.002 |
|  | CC | 19(12.0) | 286.3(226.1-346.5) |  |
| ABCB1, ATP binding cassette subfamily B member 1 | | | | |

Supplementary Table 3. Univeriate and multivariable analyses to identify predictors for time to bleeding complications related to edoxaban administration

| Predictors |  | Unadjusted HR | | Model I | Model II |
| --- | --- | --- | --- | --- | --- |
|  |  | (95% CIs) | | Adjusted HR | Adjusted HR |
|  |  |  | | (95% CI) | (95% CI) |
| Age | ≥ 65 years | 1.63(0.47-5.64) | |  |  |
| Female |  | 0.51(0.18-1.43) | |  |  |
| Prescription dose | Overdose | 6.24(1.43-27.30)^*^ | | 6.81(1.43-32.36)^*^ | 6.77(1.42-32.16)^*^ |
| rs4149057 (T>C) | CT, CC | 2.95(1.05-8.26)^*^ |  | | 2.97(1.02-8.60)^*^ |
| rs999278 (C>A) | AC, AA | 2.87(1.02-8.05)^*^ | | 2.93(1.01-8.49)* |  |
| rs2306283 (A>G) | GG | 0.37(0.13-1.05) | |  |  |
| rs4149056 (T>C) | TC, CC | 3.68(1.45-9.33)^**^ | | 3.91(1.52-10.10)^**^ | 3.90(1.51-10.05)^**^ |
| rs2417957 (C>T) | CT, TT | 0.38(0.14-1.06) | |  |  |
| *ABCB1* rs3842 *(T>C)* | CC | 4.11(1.54-10.97)^**^ | | 3.75(1.32-10.67)^*^ | 3.72(1.31-10.60)^*^ |
| Model I included variables of sex, age, prescription dose, *ABCB1* rs3842, *SLCO1B1* rs999278, rs2306283, rs4149056, and rs2417957. Model II included variables of sex, age, prescription dose, *ABCB1* rs3842, *SLCO1B1* rs4149057, rs2306283, rs4149056, and rs2417957. CI: confidence interval; OR: odds ratio. ^*^P < 0.05, ^**^P < 0.01 | | | | | |
